# F194. ASSOCIATION STUDY BETWEEN TREATMENT RESPONSE OF AMISULPRIDE AND DOPAMINE D3 RECEPTOR GENE POLYMORPHISMS

**DOI:** 10.1093/schbul/sby017.725

**Published:** 2018-04-01

**Authors:** Kwanghun Lee, Won-Myong Bahk, Bo-Hyun Yoon, Duk-In Jon, Sang-Yeol Lee, Moon Doo Kim, Beomwoo Nam, Min-Kyu Song

**Affiliations:** 1 Dongguk University Hospital; 2 Yeouido St. Mary’s Hospital, The Catholic University of Korea; 3 Naju National Hospital; 4 College of Medicine, Hallym University; 5 Wonkwang University, School of Medicine; 6 School of Medicine, Jeju National University; 7 School of Medicine, Konkuk University; 8 Yesan Mental Health Clinic

## Abstract

**Background:**

The aim of this study is to evaluate the association between rs6280 and rs905568 genetic polymorphism of DRD3 gene and the treatment response of amisulpride.

**Methods:**

After six weeks treatment of amisulpride, 125 schizophrenia patients were interviewed based on the Positive and Negative Syndrome Scale (PANSS) and the Clinical Global Impression-Severity (CGI-S). The genotyping for rs6280 and rs905568 was performed using TaqMan single nucleotide polymorphism (SNP) genotyping assay.

**Results:**

There was no significant difference in the frequency of genotype and allele of rs6280 between the responders and non-responders based on the total, positive, and general score of PANSS and CGI-S score. However, there was a significant association between this SNP and treatment response in the negative score of PANSS (χ2 = 5.23, p = 0.022). There was no significant association between rs905568 and the response in positive, negative, general, and total PANSS score and CGI-S score.

**Discussion:**

This is the first positive association study between DRD3 gene and the treatment response of negative symptoms to amisulpride in Korean schizophrenia patients. A larger scale research on more SNP of the DRD3 gene will make a progress in the study of pharmacogenetics on the treatment response of the amisulpride.

